# Why clinicians do not implement integrated treatment for comorbid substance use disorder and posttraumatic stress disorder: a qualitative study

**DOI:** 10.3402/ejpt.v5.22821

**Published:** 2014-02-05

**Authors:** Nele Gielen, Anja Krumeich, Remco C. Havermans, Feikje Smeets, Anita Jansen

**Affiliations:** 1 Mondriaan, MAIAR, Heerlen, The Netherlands; 2 Department of Clinical Psychological Science, Faculty of Psychology and Neuroscience, Maastricht University, Maastricht, The Netherlands; 3 Department of Health, Ethics & Society, Faculty of Health, Medicine and Life Sciences, Maastricht University, Maastricht, The Netherlands; 4 Department of Psychiatry & Psychology, Faculty of Health, Medicine and Life Sciences, Maastricht University, Maastricht, The Netherlands

**Keywords:** Addiction, trauma, qualitative, healthcare provider, perceptions

## Abstract

**Background:**

Healthcare providers working in addiction facilities do not often implement integrated treatment of comorbid substance use disorder (SUD) and posttraumatic stress disorder (PTSD) while there is empirical evidence to do so.

**Objective:**

This study aims to get insight into the views of clinicians with regard to the diagnosis and treatment of PTSD in SUD patients.

**Method:**

A qualitative research method was chosen. Fourteen treatment staff members of different wards of an addiction care facility were interviewed by an independent interviewer.

**Results:**

Despite acknowledging adverse consequences of trauma exposure on SUD, severe underdiagnosis of PTSD was mentioned and treatment of PTSD during SUD treatment was not supported. Obstacles related to the underestimation of PTSD among SUD patients and to the perceptions of SUD clinicians concerning the treatment of comorbid SUD/PTSD were reported.

**Conclusions:**

It is concluded that SUD facilities should train their clinicians to enable them to provide for integrated treatment of SUD/PTSD.

Posttraumatic stress disorder (PTSD) is a frequent co-occurring disorder in patients who seek treatment for their substance use disorder (SUD). Prevalence rates around 35% are mentioned in previous studies (Bonin, Norton, Asmundson, Dicurzio, & Pidlubney, 2000; Driessen et al., 2008; Gielen, Havermans, Tekelenburg, & Jansen, 2012; Ouimette, Coolhart, Funderburk, Wade, & Brown, 2007; Read, Brown, & Kahler, 2004; Reynolds, Mezey, Chapman, Wheeler, Drummond, & Baldacchino, 2005), while trauma exposure was found to be prevalent in 89–97.4% of SUD patients (Dansky, Saladin, Coffey, & Brady, 1997; Farley, Golding, Young, Mulligan, & Minkoff, 2004; Gielen et al., 2012; Read et al., 2004; Reynolds et al., 2005). SUD patients with PTSD often use substances to deal with the emotional pain caused by their trauma (Khantzian, 1997; Leeies, Pagura, Sareen, & Bolton, 2010; Ouimette, Read, Wade, & Tirone, 2010). Treatment prognosis is relatively poor in these patients (Najt, Fusar-Poli, & Brambilla, 2011; Read et al., 2004). In comparison with SUD patients without PTSD, the comorbid diagnosis of SUD/PTSD is related to a higher percentage of overdoses, suicide attempts, and more treatment days (Mills, Teesson, Ross, Darke, & Shanahan, 2005).

Over the last decade, a large body of research on the treatment of SUD patients with PTSD has accumulated (e.g., Henslee & Coffey, 2010; Hien, Cohen, Miele, Litt, & Capstick, 2004; Mills et al., 2012; Najavits, Gallop, & Weiss, 2006). Integrated treatment focusing on both disorders simultaneously seems to provide a better outcome than treatment that focuses on just one disorder at a time (Dass-Brailsford & Myrick, 2010; Mills et al., 2012; Zatzick et al., 2004). In addition to the importance to treat SUD and PTSD simultaneously, previous research also documents the need for structural assessment of PTSD in every new SUD patient who enters treatment (Gielen et al., 2012; Back, Waldrop, Brady, & Hien, 2006; Ruzek, Polusny, & Abueg, 1998). Guidelines, focusing on the PTSD/SUD comorbidity and aimed to improve the quality of healthcare, have been developed to inform clinicians about these research findings, thus bridging the gap between theory and practice (Kivlahan & Kaysen, 2012; Ruzek et al., 1998). These guidelines were also recently developed in the Netherlands (Snoek, Wits, Meulders, & Van de Mheen, 2012).

Despite the bulk of evidence and the development of guidelines, relatively few substance dependence treatment centres have implemented an integrated treatment approach, leaving PTSD in most cases untreated during SUD treatment (Glover-Graf & Janikowski, 2001; Najavits, Sullivan, Schmitz, Weiss, & Lee, 2004; Young, Rosen, & Finney, 2005). Furthermore, assessment for PTSD does not take place in every new patient, and underdiagnosis is fairly common (Gielen et al., 2012; Young et al., 2005). Although it is not clear what the specific reasons are for this contradiction between theory and practice, we do know that, in general, translation of research into practice is difficult (Forsner, Hansson, Brommels, Wistedt, & Forsell, 2010; Rothkrauf & Eby, 2011).

One of the reasons why implementation of research findings is difficult to accomplish is that individual clinicians hold different opinions about how to manage their patients (Leentjens & Burgers, 2008; Sorensen & Kosten, 2011). This explanation is related to Arthur Kleinman’s (1980) theory. According to Kleinman’s explanatory model (EM) approach, every individual, professionals and patients alike, hold different beliefs about a particular illness. These beliefs, or “Ems,” are shaped by an individual’s beliefs and impact how this individual applies these beliefs in particular illness episodes. Kleinman (1980) identified five constructs that determine how an individual defines and approaches a (health) problem and that constitute an individual’s EM. These constructs include: (1) notions about the aetiology of the illness, (2) ideas about symptom onset, (3) views about the pathophysiology of the illness, (4) perceptions about the course of illness, and (5) the recommended treatment. Kleinman states that how an individual defines a certain illness influences how this illness will be dealt with (i.e., what kind of treatment one thinks appropriate and by whom and what kind of assessment criteria one applies to these actions, including notions of what can be expected of professionals in terms of practices, attitude, and responsibilities). Kleinman focuses on the distinction between EMs of professionals and EMs of patients and how the discrepant beliefs influence the treatment of a certain disease. His model can also be used to study the perception of professionals of a particular health problem such as SUD/PTSD, their attitude regarding existing or new treatment protocols and procedures, the kind of criteria they use to assess effectiveness and quality of existing or new practices and procedures, their actual evaluation of procedures and protocols and the preparedness to adopt new treatment regimes.

This article focuses on clinicians’ EMs of comorbid SUD and PTSD. To understand why substance abuse clinicians do not implement evidence-based integrated treatment for patients with SUD/PTSD, it is useful to get insight into their views. The goal of this study is to explore why healthcare providers working in the addiction field do not offer integrated treatment for SUD/PTSD. We also aim to formulate implementation guidelines for addiction facilities. Since this is a field study of clinician’s perceptions, a qualitative research method is the most suitable. The EM approach will be used as a tool to identify points of improvement.

To our knowledge, this is the first qualitative study that explores the perceptions of SUD clinicians about how to treat patients with comorbid SUD/PTSD patients. Some researchers have previously used surveys with the same goal. Glover-Graf and Janikowski (2001) and Janikowski and Glover-Graf (2003) used the SACSCIH to survey substance abuse clinicians who work with victims of incest. Najavits, Norman, Kivlahan, and Kosten (2010) and Young et al. (2005) used surveys (resp. the VA version of the Clinician Survey on PTSD and Substance Abuse and a survey developed by the authors) in VA (Veterans Affairs) settings to learn more about the screening, treatment, and referral of SUD/PTSD patients. While these studies focused on different subjects (clinicians in VA settings and SUD clinicians working with victims of incest), in two other studies clinicians were surveyed about the treatment of SUD/PTSD. Najavits (2002) surveyed 147 clinicians using the Clinical Survey on PTSD and Substance Abuse and concluded that the treatment of SUD/PTSD was rated as more difficult to treat than either disorder alone. Interestingly, she also found that clinicians perceived more gratification than difficulty in working with this subgroup of patients. Back, Waldrop, and Brady (2009) tested 423 clinicians using the same survey as Najavits (2002). They found that the issue on when and how to integrate the treatment of SUD and the treatment of PTSD was perceived as the most challenging.

In this exploratory study, we made use of a topic list and specifically addressed the following themes: assessment of trauma, diagnosis of PTSD and treatment of PTSD. By use of semistructured qualitative interviews, clinicians were asked about these topics in order to gauge the clinician’s EM of SUD/PTSD. We questioned the current procedures, responsibilities, and possible obstacles.

## Methods

### Design and justification

Since this study aimed to explore the individual perceptions of substance abuse therapists, we used a qualitative research method. Semistructured in-depth interviews with open-ended questions were used. This research method best fitted the current exploratory research questions.

### Data collection and sampling

The current research took place in the addiction care division of Mondriaan. Mondriaan is a large institution with different certified treatment centres in the whole region of southern Limburg in the southern part of the Netherlands (total population currently estimated at 607,000). One of these centres is a large substance/behavioural dependence treatment facility. Staff members of different wards of this facility were included in this study. A purposeful sampling strategy was used to achieve a representative sample with work setting, position, and years of experience as selection criteria. These criteria were chosen because of their assumed influence on a clinician’s EM. A profile list of possible participants in terms of these selection criteria was made, and each ward was contacted to find participants with the desired profile.

A total of 20 candidate participants received an email explaining the goals and procedure of the study. Two persons declined participation due to time restrictions and three were non-responders, leaving 15 staff members who were eventually interviewed. Owing to technical problems, one interview could not be transcribed. Since a suitable saturation level was reached after 14 interviews, non-responders were not replaced in this study. The characteristics of the sample are outlined in Table 1.

**Table 1:** Sample characteristics

	Work setting	Position	Experience
1	MC/FPAC/DDW	Psychotherapist	>5 years
2	CCW	Family system therapist	>5 years
3	FPAC	Psychiatric nurse	<5 years
4	IT	Social worker	<5 years
5	DDW/MC	Psychologist	<5 years
6	CCW/MC	Psychiatrist	>5 years
7	AT	Psychologist	>5 years
8	IT/AT	Psychiatrist	<5 years
9	CCW	Psychiatric nurse	<5 years
10	IT, AT	Social worker	>5 years
11	CCW	Psychologist	>5 years
12	AT	Social worker	>5 years
13	DDW	Psychologist	<5 years
14	CCW/MC	Unit manager	>5 years

Work setting: IT, intake team; CCW, clinical continuation ward; FPAC, forensic psychiatric addiction care; DDW, double diagnosis ward; MC, motivational centre; and AT, ambulatory treatment.

### Procedure

From October 2008 until January 2009, the selected staff members were interviewed. To prevent bias, an independent trained interviewer questioned the participants (investigator triangulation). All participants provided informed consent and agreed that the interviews would be recorded on audiotape. After an ice-breaker opening question (“Can you tell me something about the procedure when a new patient enters treatment?”), a topic list was used to cover the formulated themes: assessment of trauma, diagnosis of PTSD, and treatment of PTSD. We were interested in the current procedure, the responsibilities, and possible obstacles. The interviewer, who was familiar with the organization, was instructed to ask open-ended questions and to approach the respondents with a natural curiosity and respect to ensure honest and frank answers. Further instructions included holding a natural fluency in the questions and to communicate clearly. When a participant gave answers outside the scope of the interview, the interviewer brought the conversation back to the subject. Examples of questions were: (1) At what moment in the treatment process do you refer patients for PTSD treatment? (2) What treatment do you judge to be ideal for patients with SUD and PTSD? (3) What kind of tools do you use to diagnose PTSD? or (4) Who is, according to you, responsible to question patients about past trauma?

Frequent debriefing sessions were organized to optimize the quality of the interviews. To further improve the reliability of the results, we used member checking: the interviewer was instructed to restate or summarize the answers of the respondent and then to question the respondent to determine accuracy. Each interview lasted between 15 and 30 min. An independent coworker made transcripts of each interview.

### Data analysis

The transcripts were analysed using content analysis. We chose to categorize the data with inductive analysis. Pope, Ziebland, and Mays (2000) describe this procedure in their article. We will now give a detailed description of how we analysed our data. The transcriptions of the interviews were read, and first marginal notes were added. Initially, these were open codes. Whatever came up that suited the text segment was written in the margin. When this was done for all the interviews, sensitizing concepts were chosen, reflecting associations of marginal notes between the interviews. The following selective codes were created: (1) definition of comorbid SUD/PTSD and assumptions about the underlying cause, (2) suitable treatments, (3) responsibilities, (4) anamnestic phase, (5) diagnosis process, and (6) preconditions.

For each interview, a new document was composed with the six concepts as headings, and the exact copies of the respective text fragments were copy-pasted below. Summaries of the text fragments were made, each fragment resulting in a one- or two-sentence summary. These summaries were then combined for all interviews, resulting in six documents with all summaries for each theme. The summaries were carefully checked for connections, and a higher level of abstraction was reached with new categories. After that, a fluent text was written for each category with the short summaries serving as illustrations. When this was done for the six concepts, a presentation was prepared and by doing so, we came up with a new and clearer way to organize the results.

Concept 3 (responsibilities) and 6 (preconditions) could be merged in the other four concepts. Furthermore, it became clear that, besides the first concept (definition of illness), a subdivision in (a) “current situation,” (b) “ideal/ desired situation,” and (c) “needs to achieve the desired goals” was suitable for the remaining three themes. Finally, during analysis, subdivisions (b) and (c) were combined since these constructs were closely connected with each other. The final categories thus emerged as (1) definition and aetiology of comorbid SUD/PTSD, (2) anamnestic phase, (3) diagnosis process, and (4) suitable treatments. These categories match with Kleinman’s EM constructs, with the first category corresponding to Kleinman’s aetiology/course of illness/symptom onset and pathophysiology EM constructs and categories 2–4 belonging to Kleinman’s recommended treatment EM domain.

To account for a potential researcher effect, the transcribed interviews were independently analysed by two different investigators (investigator triangulation). Besides the analysis which is outlined in detail in this section, the fourth author analysed the information in another way: she sorted the interviews by treatment facility and made four subcategories: diagnostics, treatment, referral and other relevant notions. The main relevant topics for each facility were selected and final summaries were compared between treatment units to identify issues that pertained to the entire organisation. Although the two investigators used different analysing techniques, they did come up with comparable results and conclusions (Smeets, 2009).

## Results

### Definition and aetiology of comorbid SUD/PTSD

When we asked the interviewees to estimate the prevalence of trauma exposure and PTSD in their SUD caseload, the opinions differed quite a lot, with estimates of trauma exposure ranging between 0.5 and 100%.

Trauma, as in PTSD in the DSM, is rare. Maybe only 1% or even less.

I assume that every patient who is treated here has ever been exposed to a traumatic experience.

Only one interviewee (a psychologist) mentioned an indirect estimate of PTSD in SUD patients and stated to have never met a SUD patient with PTSD. No other interviewees reported specific estimations about PTSD prevalence.

I’ve never seen real PTSD. So, its prevalence is quite low.

Holding the literature findings on prevalence rates of PTSD and trauma exposure in SUD patients in mind, we can conclude that the estimated prevalence rates (despite the wide range) in this study suggest a severe underestimation of the problem.

An important issue was how participants interpreted comorbid SUD/PTSD. Among the interviewees many views came up. One of these corroborated the self-medication theory. According to the clinicians that referred to this theory, traumatized individuals use substances to numb negative feelings or to suppress intrusions. As a consequence, these patients never learn to handle their problems and their symptoms become chronic. In this interpretation, PTSD symptoms are, in other words, understood as a mediating factor leading to craving and possible relapse.

Other interpretations were offered. One of these suggested that SUD patients often expose themselves to dangerous or trauma-prone environments (high-risk hypothesis; Stewart & Conrod, 2008). It was assumed that when this trauma exposure happens after initial substance use, substance use aggravates.

And, indeed, you meet severely addicted people who often expose themselves to dangerous situations which increases the risk for trauma.

Another interpretation referred to the neurobiological dimension of substance dependence. This interpretation referred to a preprogrammed biological vulnerability to develop a mental disorder (diathesis stress model; Roberts, Moore, & Beckham, 2007). In interpretations referring to the neurobiological model, it was asserted that early trauma can disturb neurobiological systems (reward system, neurotransmitters, and sleep pattern), leading to different effects after substance intake and putting someone at risk for the development of a SUD (disturbed stress reaction hypothesis).

A final interpretation focused on an interaction between trauma, SUD, and personality. Trauma exposure, it was asserted, results in a change in personality or even in the development of a personality disorder and this can make a person vulnerable for the development of an SUD. Personality, in this definition, can be understood as a mediating factor.

These definitions have a presumed relationship between PTSD and SUD in common. No matter how they perceived the causality, participants agree that it is important to have knowledge about the trauma as it can increase understanding the patient’s motivations contributing to his/her SUD. The clinicians also agree that PTSD symptoms make it particularly difficult for the SUD patient to get clean or sober.

The first main finding is that the majority of clinicians seem to have a reasonably good understanding about the interrelatedness between SUD and PTSD. Although the clinicians severely underestimate the prevalence of PTSD in their patients (Gielen et al., 2012; Reynolds et al., 2005), their ideas about the aetiology, symptom onset, pathophysiology, and course of illness are in accordance with the literature (Langeland, 2009; Roberts et al., 2007; Stewart & Conrod, 2008). The fact that clinicians refer to existing theoretical models, including the self-medication theory, the high-risk hypothesis, the diathesis stress model, and the disturbed stress reaction hypothesis, adds to the conclusion that the interviewed clinicians are well aware of the negative influences of trauma exposure and PTSD symptoms on SUD. One would thus expect clinicians to take PTSD into account during SUD treatment. But that is clearly not the case. Why not?

### Trauma anamnesis in SUD patients

#### Current situation

According to the interviewees, there is a more or less standard screening at intake of any patient. This screening interview typically includes questions about current symptomatology, family history, life history, etc. Although trauma exposure is not specifically enquired in this anamnesis, it does, however, often suggest itself to the attentive intaker. The clinicians notice that sometimes patients spontaneously report trauma exposure. When trauma is considered by the clinician, it is done very carefully and only superficially.

But to really ask deeper about the trauma is of course not done.

When a patient enters treatment, clinicians usually rely on intake reports (and/or on existing patient files) and do not ask about possible trauma. It is noted that patients who are known for years sometimes have an unknown case-history. Furthermore, the interviewed intake clinicians agreed that sometimes they decide not to enquire about trauma because of the delicacy of the matter. The trauma anamnesis is implicitly expected to be continued during later treatment.

When I notice that they (the patients) find it (the trauma anamnesis) very hard, then I find it quite a challenge to ask about trauma. They often don’t see me anymore after the intake. So, I then decide not to go into it and leave it for the treatment phase.

The opinions on the responsibility to ask about trauma history are mixed. The trauma anamnesis should be done by the intake clinician, by the psychologist concerned, by the case manager or by the individual mentor (in case of hospitalization). Another view is that anyone should do it.

With regard to the anamnesis of trauma exposure we can conclude that: (1) trauma exposure is not directly questioned in new patients, and intake clinicians do not use specific validated assessment tools, (2) clinicians seem to favour a very careful approach with regard to the trauma anamnesis, (3) in the case of already known patients clinicians rely on former and possibly outdated patient files, and finally, (4) the responsibility to enquire about trauma is indistinct. Therefore, it is clear that there is a lack of a protocol concerning the trauma anamnesis in SUD patients. It is possible that the previously mentioned underestimation of trauma exposure prevalence is related to this absence of trauma anamnesis protocol. Since most clinicians believe that PTSD is not a frequent problem in SUD treatment, they do not experience a need for a protocol to assess trauma. The cautious approach towards trauma, on the other hand, may reflect the supposed association between talking about trauma, increase of PTSD symptoms and consequently an increase in craving and possible relapse of addictive behaviour. This thinking contrasts clinical guidelines (e.g., Gielen et al., 2012; Ruzek et al., 1998) and the scientific literature. McHugo and colleagues (2005) interviewed over 2,700 SUD patients with co-occurring mental disorders about how trauma assessment was tolerated. The results of McHugo’s study indicated that the assessment was not only well tolerated, but was even regarded as a positive experience by most patients.

#### Ideal—desired situation

When the interviewer specifically asked for the need to assess trauma during intake or treatment, the clinicians do agree that trauma anamnesis is essential and they add that every patient should be directly questioned about possible trauma. They also state that is very important to clearly report these facts in their patient files and to be aware that already known patients may have incomplete or out-of-date patient files.

But there are people who are for instance on methadone and have been in and out of treatment for over 10 years. Not a lot of attention is paid to their history and who knows what further lurks beneath the surface.

Interviewees state that when trauma is not questioned during intake, the intake clinician should clearly communicate this with the responsible substance abuse clinician.

In contrast to current practice, the desired ideal situation is in line with the guidelines and can lead to a better assessment of trauma exposure. Interviewees provide some suggestions on how to reach this goal. The responsibility to assess trauma should be defined more clearly and training on how to assess trauma is needed for the intake clinicians.

### Diagnosing PTSD in SUD patients

#### Current situation

Clinicians emphasize that it is important to diagnose PTSD. The following procedure in the diagnosis process of a new patient is reported. First, the intake clinician, often a social worker or a psychiatric nurse, makes a temporary diagnosis based on an interview. In case of uncertainty, the patient is then referred to a psychiatrist. This psychiatrist only sees a minority of the patients and does not use any standardized assessment tools in reaching a diagnosis.

They (the patients) rarely end up with me (the psychiatrist) … I have 12 hours for the treatment of 1000 patients. I expect there are a lot of patients among them that have a whole lot of (psychiatric) problems of which I’m not aware.

When the temporary diagnosis does seem clear, the patient is discussed in a multidisciplinary team meeting, including a psychologist and a psychiatrist. During this discussion, a final diagnosis is made. The psychiatrist holds the end responsibility to make a diagnosis.

It is acknowledged that, in case of previously known patients, the diagnoses of the old patient files are often used without further inquiry.

The interviewees indicate that during treatment the diagnosis is malleable. Team members sometimes signal trauma-related symptoms in a patient and then refer the patient for further consultation to the psychiatrist or resident psychologist. This may result in a change in the patient’s diagnosis. Again, both the resident psychiatrist and psychologist rarely use standardized assessment tools to reach a specific diagnosis. As for PTSD specifically, the interviewees mention that no specific PTSD questionnaires or interviews are available or known to them. This of course may result in misdiagnosis or as in the case of PTSD, severe underdiagnosis. Indeed, the interviewed clinicians are in agreement with regard to the severe underdiagnosis of PTSD in their patient group.

I think our patient group is heavily underdiagnosed.

Finally, the interviewees state that diagnosis can be difficult in SUD patients because of the similarity between PTSD symptoms and addiction related symptoms (e.g., intoxication and withdrawal).

It is difficult to disentangle PTSD and addiction symptoms. What is what?

#### Ideal—desired situation

The necessity to screen for PTSD in every patient at different times (since a diagnosis can change over time) during treatment is stressed. Furthermore, clinicians are aware that they should be more alert for PTSD symptoms. Although the importance of diagnosing PTSD in SUD patients is recognized, three important difficulties can be indicated. Firstly, the clinicians describe only two evaluation moments in which PTSD can be evaluated and report that in already known patients their previous diagnosis is copied, not re-assessed. Different existing clinical guidelines suggest at least three separate evaluation moments in all patients and a continuous monitoring of symptoms (Schatzberg, Weiss, Brady, & Culpepper, 2008; Snoek et al., 2012).

A second problem is that clinicians at intake reach a diagnosis relying solely on an interview in which PTSD or trauma exposure is not explicitly questioned. Clinicians seem to rely heavily on their clinical judgement when diagnosing PTSD in SUD patients. The combination of the reported underestimation of PTSD prevalence and the inherent bias of clinical judgement (Dawes, Faust, & Meehl, 1989; Garb, 2005) make for a plausible explanation of the underdiagnosis of PTSD.

The third difficulty is that PTSD symptoms can be confused with intoxication and withdrawal symptoms. Although some SUD related symptoms mimic or overlap with PTSD symptoms (e.g., sleep disturbance, difficulty concentrating, feelings of detachment, irritability), PTSD is characterized by unique criteria: the exposure to a criterion A event and intrusive trauma-related symptoms (4th ed., text rev.; DSM–IV–TR; American Psychiatric Association, 2000). To overcome this problem of symptom confusion, the assessment of PTSD is best done after a period of abstinence (Snoek et al., 2012).

### PTSD treatment in SUD patients

#### Current situation

The interviews with the clinicians make it clear that the treatment facility promotes sequential treatment in which SUD is treated before PTSD. The focus should be on SUD and patients are sometimes advised not to go into trauma therapy during SUD treatment.

I usually gave the advice that trauma therapy was not an option at that moment.

Doing that, you may cause more misery than necessary. In cases like that I’m more inclined to cover the trauma with sand, a concrete layer, and to continue work upon that rotten foundation.

Although the sequential view on the treatment of comorbid SUD and PTSD is seen as outmoded by different interviewees, quite a few interviewees strongly argue against simultaneous treatment. To treat PTSD during SUD treatment is seen as too soon, harmful, counterproductive, unwise, and distracting.

Sometimes doing nothing is less harmful.

The real trauma therapy is not done here because it usually impedes addiction treatment.

In contrast with this current practice and thinking, the guidelines are very clear: integrated treatment is the standard of care (Kivlahan & Kaysen, 2012; Mueser, Noordsy, Drake, & Fox, 2003; Snoek et al., 2012).

Despite the fact that guidelines emphasize using pharmacotherapy only as an additional form of therapy (Snoek et al., 2012), the interviewed clinicians indicate that pharmacotherapeutic treatment is the current treatment of choice for PTSD in SUD patients. Interviewees state that neuroleptica and Selective Serotonin Reuptake Inhibitors (SSRI’s) are prescribed for PTSD in SUD patients, leading to a decrease of intrusions and other PTSD symptoms. Psychopharmaceuticals are also advised for trauma-related sleeping disorders.

Clinicians favour a present-focused approach for PTSD treatment above a trauma- or past-focused treatment. This approach involves carefully exploring the impact of trauma, developing new coping techniques, focusing on the future, psycho-education, symptom reduction, and increasing stability. Interviewees state the importance of not talking about the trauma with the patient and to restrict or refer patients when they do.

In essence, you don’t discuss the trauma.

The interviewees note that in patients with more introspective skills, the psycho-education can be further deepened to increase the understanding and to link trauma-related patterns with addiction. Therapy can then include social skills training or rational emotive therapy.

According to the interviewees, all team members, under the supervision of a psychologist or psychiatrist, can apply the above-mentioned approach. There should, however, be a match between psychopathology and expertise.

Current research suggests that past-focused therapies, not present-focused therapies, should—even in SUD patients—be the treatment of choice (Berenz & Coffey, 2012; van Dam, Vedel, Ehring, & Emmelkamp, 2012).

The arguments that are given against trauma therapy during SUD treatment suggest that clinicians favour a sequential, pharmacotherapeutical, or present-focused treatment approach for PTSD, because they believe that a past-focused integrated approach might exacerbate PTSD symptoms promoting drug or alcohol craving and possible addictive behaviour relapse.

#### Ideal—desired situation

Although sequential treatment seems to be the current way of handling SUD/PTSD patients, clinicians do report that dividing the treatment of SUD and PTSD is artificial and difficult to bring into practice. Because of the high suffering of the patient and the close link between PTSD symptoms and relapse, both disorders should be treated simultaneously. The interviewees recognize the ethical responsibility to do a co-treatment of SUD and PTSD.

… so we have the responsibility to treat everything.

According to the interviewees, this simultaneous treatment should, ideally, be given intramurally in a double diagnosis ward where a long enough admission time is possible.

But, of course, it’s not always relevant. It depends on where, on which department, and with what goal, someone is hospitalized.

If PTSD treatment takes place on an ambulatory basis, an emergency admission should be possible in case of severe psychological deterioration of the patient. Clinicians further express the need to have a treatment protocol for comorbid SUD/PTSD. In this protocol, special attention should be given to a no show procedure as it is expected that no shows may occur more often with this subgroup of patients. A clear and uniform view of the institution about how to deal with comorbid SUD/PTSD is another important condition that is highlighted. If the institution stimulates PTSD treatment during SUD treatment, more time and money should be reserved for this aim.

Not much is done with that information (PTSD diagnosis) because we can’t do so much with it, because we don’t have the means.

Training and supervision is needed in order to increase the knowledge about PTSD and about the impact of PTSD treatment. Intervision is suggested to prevent clinicians from developing secondary PTSD.

We can’t actually do that much with trauma within our departments of addiction care. We do not have the time or the expertise.

As in the previous sections, there is an explicit contrast between the current practice and the situation that is described as ideal. The suggestions that are provided offer good possibilities to alter the current practices towards a more evidence-based treatment. Lack of time, money and expertise are mentioned as reasons why integrated treatment is currently not offered to SUD/PTSD patients.

### Gap between theory and practice

Almost every interviewee (12 out of 14) believes that trauma and SUD are interrelated. Most clinicians report that substance use is negatively influenced by PTSD symptoms or that substance use follows trauma exposure. According to Kleinman’s model, these statements should predict that treatment of PTSD takes place during SUD treatment. However, nearly all clinicians admit that PTSD treatment does not occur during SUD treatment.

Interviewee 1: It (trauma/PTSD) can be a maintaining factor (for addiction).Sometimes doing nothing is less harmful.

Interviewee 2: Trauma is always of influence.

I don’t think you should work on trauma processing here. That’s more something for after the treatment (of addiction).

Interviewee 4: The real problems start when someone is going to detoxify … then the sorrow, the pain and the grief emerge.

We can’t do that much with trauma … During treatment we thus only focus on substance use.

## Discussion

In order to understand why substance abuse clinicians do not implement evidence-based integrated treatment for patients with SUD/PTSD, the current report explored how healthcare providers define the comorbid disorders and how their perceptions influence clinical practice. The purpose of this study was to unearth perceptions and practices regarding SUD/PTSD in order to improve implementation of best practice guidelines concerning the comorbidity of SUD and PTSD for addiction facilities.

This study suggests that two factors affect the underdiagnosis and undertreatment of PTSD in SUD facilities, which is also reported in the literature (Gielen et al., 2012; Glover-Graf & Janikowski, 2001; Najavits et al., 2004; Young et al., 2005). (1) Although most clinicians are well aware of the adverse consequences of trauma exposure and PTSD on SUD, in general, SUD clinicians are not aware of the high prevalence rates of trauma exposure and PTSD among their patients. As a consequence, PTSD is not a priority and adequate treatment protocols, specific diagnostic tools and even the clinical guidelines are not well known. (2) Clinicians believe that talking about past traumas elicits craving and possible relapse. This belief leads to a too-careful approach or no approach of past trauma.

The fact that the interviewees advised specific improvements about the trauma anamnesis, PTSD diagnosis, and PTSD treatment, which resembled the SUD/PTSD guidelines, suggests that their theoretical knowledge might not be the most important reason for not following the clinical guidelines. The main hurdles appear to be practical: lack of time, money, and expertise. It should be noted though that the interviews forced the clinicians to think about PTSD in SUD patients. This impromptu awareness of PTSD in their patients may have led them to stress the importance of diagnosing and treating PTSD in SUD patients. Nonetheless, with regard to Kleiman’s EM approach, we can conclude that the views held by SUD clinicians about PTSD prevalence and the supposed negative influence of discussing trauma certainly affect how the clinician handles comorbid PTSD in SUD patients. The matter is, however, further complicated by the lack of means.

Unfortunately, we could not ask participants to determine the accuracy of the conclusions because too much time passed between the interviews and the analysis. Nonetheless, the present findings might provide important implications for SUD treatment facilities.

## Conclusions

The results of this study confirm that PTSD treatment was not a focus during SUD treatment. Although clinicians were well aware of the adverse consequences of trauma exposure and PTSD on SUD, hindrances related to the underestimation of PTSD in SUD patients, a too-careful approach and lack of time and money prevented an adequate diagnosis and treatment of PTSD. SUD facilities should therefore invest in the evidence-based integrated approach of comorbid SUD/PTSD. The results of this study corroborate previous findings that indicate that SUD treatment facilities have a lot to gain by investing in integrated/simultaneous SUD/PTSD treatment. Clinicians should be educated and trained to be able to assess PTSD/trauma, using reliable and valid measures, and to provide evidence-based SUD/PTSD treatment. Since successful implementation is also dependent on the perceptions SUD/PTSD patients hold, future research might focus on this topic.
